# Optimization of Sample Preparation for the Identification and Quantification of Saxitoxin in Proficiency Test Mussel Sample using Liquid Chromatography-Tandem Mass Spectrometry

**DOI:** 10.3390/toxins7124853

**Published:** 2015-11-25

**Authors:** Kirsi Harju, Marja-Leena Rapinoja, Marc-André Avondet, Werner Arnold, Martin Schär, Stephen Burrell, Werner Luginbühl, Paula Vanninen

**Affiliations:** 1 VERIFIN (Finnish Institute for Verification of the Chemical Weapons Convention), Department of Chemistry, University of Helsinki, P.O. Box 55, A. I. Virtasen aukio 1 FI-00014, Finland; marja-leena.rapinoja@helsinki.fi (M.-L.R.); paula.vanninen@helsinki.fi (P.V.); 2 Federal Department of Defence, Civil Protection and Sport, SPIEZ LABORATORY, Austrasse 1, Spiez CH-3700, Switzerland; marc-andre.avondet@babs.admin.ch (M.-A.A.); werner.arnold@babs.admin.ch (W.A.); martin.schaer@babs.admin.ch (M.S.); 3 Marine Institute, Marine Environment and Food Safety Services, Rinville, Oranmore, Co. Galway, Ireland; stephen.burrell@marine.ie; 4 ChemStat, Aarstrasse 98, Bern CH-3005, Switzerland; info@chemstat.ch

**Keywords:** paralytic shellfish poisoning toxins, saxitoxin, liquid chromatography-mass spectrometry, mussel

## Abstract

Saxitoxin (STX) and some selected paralytic shellfish poisoning (PSP) analogues in mussel samples were identified and quantified with liquid chromatography-tandem mass spectrometry (LC-MS/MS). Sample extraction and purification methods of mussel sample were optimized for LC-MS/MS analysis. The developed method was applied to the analysis of the homogenized mussel samples in the proficiency test (PT) within the EQuATox project (Establishment of Quality Assurance for the Detection of Biological Toxins of Potential Bioterrorism Risk). Ten laboratories from eight countries participated in the STX PT. Identification of PSP toxins in naturally contaminated mussel samples was performed by comparison of product ion spectra and retention times with those of reference standards. The quantitative results were obtained with LC-MS/MS by spiking reference standards in toxic mussel extracts. The results were within the z-score of ±1 when compared to the results measured with the official AOAC (Association of Official Analytical Chemists) method 2005.06, pre-column oxidation high-performance liquid chromatography with fluorescence detection (HPLC-FLD).

## 1. Introduction

Paralytic shellfish poisoning (PSP) toxins are highly toxic compounds produced by marine dinoflagellates and freshwater cyanobacteria. Structures and relative mouse toxicities of selected PSP toxins are given in Table 1. 

**Table 1:** The structures and relative toxicities of selected paralytic shellfish poisoning (PSP) toxins [1].

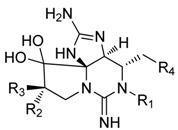

Compound	R_1_	R_2_	R_3_	R_4_	Relative toxicity
Saxitoxin (STX)	H	H	H	OCONH_2_	1.0000
Decarbamoyl saxitoxin (dcSTX)	H	H	H	OH	0.5131
Neosaxitoxin (NEO)	OH	H	H	OCONH_2_	0.9243
Gonyautoxin 1 (GTX1)	OH	H	OSO_3_H	OCONH_2_	0.9940
Gonyautoxin 2 (GTX2)	H	H	OSO_3_H	OCONH_2_	0.3592
Gonyautoxin 3 (GTX3	H	OSO_3_H	H	OCONH_2_	0.6379
Gonyautoxin 4 (GTX4)	OH	OSO_3_H	H	OCONH_2_	0.7261
Gonyautoxin 5 (GTX5)	H	H	H	OCONHSO_3_H	0.0644

Accumulation of the PSP toxins in the food chain causes a significant health risk to people and affects the seafood industry. PSP toxins block sodium channels, and cause neurological symptoms such as numbness, muscular weakness; respiratory paralysis, and the intoxication may even lead to death [2]. Saxitoxin (STX) is highly poisonous, and the oral LD_50_ for humans is 5.7 µg/kg [3]. The mouse bioassay (MBA) was the first available official method for screening PSP toxins in mussel samples [4]. It suffers from poor sensitivity and precision, and the replacement of ethically questionable mouse bioassay with other techniques has been under discussion. High performance liquid chromatography with fluorescence detection (HPLC-FLD) and pre-column [5] or post-column oxidation [6] are now available as Association of Official Analytical Chemists (AOAC) official methods for mussel samples in addition to the MBA method and these methods have been tested in interlaboratory studies [7,8,9]. Moreover, liquid chromatography-mass spectrometry (LC-MS) methods have been used for the analysis of PSP toxins in mussel samples [10,11,12,13,14,15], but these methods are not yet accepted as official AOAC (Association of Official Analytical Chemists) methods for the analysis of PSP toxins in mussel samples. Recently, an improved sensitive and selective UPLC-MS/MS method for PSP toxins was described by Boundy *et al.* [16]. 

For the food industry, it is essential to prevent consumers from exposure to harmful toxins. Additionally, STX is a Schedule 1 chemical on the OPCW (Organisation for the Prohibition of Chemical Weapons) list of the Chemical Weapons Convention (CWC) and it has been considered to be a potential bioterrorism risk [17]. The reliable identification of STX in various matrices is necessary and the identification of the CWC related chemicals must be based on at least two different analytical techniques. Mouse bioassay and immunoassay methods are not intrinsically suitable for this purpose due to the lack of specificity for STX and possible cross-reactions with other PSP toxins. The main emphasis of the research was on the reliable identification of STX. The selection of STX analogues was based on the most common naturally occurring PSP toxins, which were also available as reference standards. The selected PSP toxins were closely related to saxitoxin and they had various substituents such as carbamoyl, hydroxyl, sulfate, and *N*-sulfocarbamoyl with different charge states and varying relative toxicities (Table 1).

The official AOAC methods utilize either 0.1 M hydrochloric acid (MBA, post-column oxidation FLD) or 1% acetic acid (pre-column oxidation FLD) in the extraction of PSP toxins from mussel samples. The heating step is essential for the proper extraction of the PSP toxins from mussel samples, but it may cause some degradation of labile PSP toxins to more toxic analogues [18]. The extraction methods have been extensively studied during the validation of the AOAC methods. Mild extraction conditions with 0.2 M acetic acid (AcOH) and no heating were recommended in the first interlaboratory studies of pre-column oxidation FLD method [19,20], though 0.1 M hydrochloric acid (HCl) was used in the original pre-column Lawrence method [21]. Later, the extraction procedure in the official pre-column oxidation method was changed to heating with 1% AcOH [5]. The appropriate extraction solvent was again discussed when developing the post-column oxidation FLD method. Post-column oxidation method has also been described with 1% acetic acid extraction [22], but, later, hydrochloric acid was found to be more suitable compared to acetic acid because of better toxin recovery, better suitability for protein precipitation with trichloroacetic acid (TCA), and similarity to mouse bioassay method. These factors led to the decision to use 0.1 M hydrochloric acid as the extraction solvent for post-column HPLC-FLD method [23,24]. Extraction of mussel samples in 80% acetonitrile (ACN) acidified with 0.1% formic acid has been applied for LC-MS/MS analysis [12,13]. 

Here, we describe an LC-MS/MS method optimised for the identification and quantification of STX and some selected PSP analogues in mussel samples. The studied method has been previously validated for algal samples [25]. Sample extraction and purification methods were now optimized for the reliable identification and quantification of STX in mussel samples by LC-MS/MS. STX identification in mussel samples was based on the OPCW retention time criteria [26] and tandem mass spectrometry (MS^2^) fragmentation pattern of the analyte when compared to certified reference standard according to the EU criteria [27]. The developed method was applied and tested in the STX proficiency test (PT) of the EQuATox project (Establishment of Quality Assurance for the Detection of Biological Toxins of Potential Bioterrorism Risk) under the 7^th^ European Union Framework Programme for Research (FP7) [28]. Materials and methods are detailed described in the experimental Section 3. 

## 2. Results and Discussion

The EQuATox project consisted of four separate PTs on four different toxin types: ricin, saxitoxin (STX), staphylococcal enterotoxin B (SEB), and botulinum neurotoxin (BoNT). In the STX PT the participating laboratories were offered the possibility to assess their performance regarding the identification and quantification of STX in four different naturally contaminated sample types: three parallel homogenized mussel samples, three parallel algal samples and two algal extracts containing PSP toxins. Additionally, the other PSP toxins were asked to be identified and quantified, if possible, under the limited test time of four weeks. In the STX PT the intention was to provide participating laboratories an overview and evaluation of existing methods for screening, analysis and identification of STX and some of its analogues and compare the results to the assigned values. 

The purpose of the LC-MS/MS method development was to optimize the sample preparation so that saxitoxin, which is listed in the Schedule 1 of the CWC, could be verified in mussel samples. The optimization of the mussel sample extraction was monitored from the saxitoxin point of view. According to the OPCW, reliable unambiguous identification of an analyte should be based on at least two independent analytical methods and the analyte has to fill the retention time and mass fragmentation criteria. The method developed first for STX was then applied for the identification of other PSP toxins in the mussel samples, and those analytes, which were clearly above the detection limits, were also quantified with the LC-MS/MS method. 

### 2.1. Optimization of the Sample Preparation

#### 2.1.1. Comparison of Extraction Solvents

**Figure 1:**
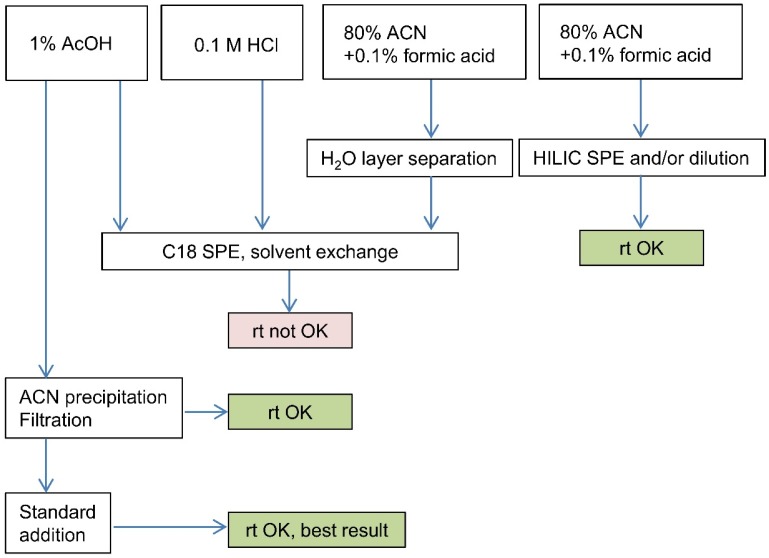
Extraction and purification scheme for the mussel samples, comparison of the retention time (*rt*) to an STX reference standard. Retention time criterion of |Δ*rt*| ≤ 0.2 min was applied [26].

In this work, parallel homogenized mussel samples prepared for the STX PT from toxic Spanish *Mytilus galloprovincialis,* toxic Canadian *Mytilus edulis* and blank Irish *Mytilus edulis* mussel were utilized in the development of the LC-MS/MS method. The total PSP toxicity of the mussel sample was set to about 1000 µg STXeq/kg, which was above the regulatory limit 800 µg STXeq/kg. Several sample preparation techniques were tested and the general sample preparation scheme is presented in Figure 1. The identification of STX was based on the comparison to a certified reference standard. Three extraction solvents, 1% AcOH, 0.1 M HCl, and 80% acetonitrile with 0.1% formic acid were compared for the preparation of mussel extracts for LC-MS/MS analysis. The obtained STX results were compared to the values measured with pre-column oxidation HPLD-FLD (126–131 ng/g). The extraction procedures for each solvent were similar, except no heating was applied for the acetonitrile extraction due to the low boiling point of acetonitrile. The separation of the water layer by freezing the 80% acetonitrile extract before further solid phase extraction (SPE) purification was tested with a slightly modified procedure described by Sayfriz *et al.* [12], but the STX recovery was low. In the preliminary extraction studies, the highest recoveries for STX were obtained with 1% acetic acid extraction. Compared to the recovery for STX with hydrochloric acid extraction, which was 20%–50%, the water-layer separated from acetonitrile extract contained less than 20% of STX. The phase separation of water from acetonitrile is difficult to optimize because these solutions are miscible at room temperature and the separation of the layers is complicated. In further studies, the acetonitrile-based extractions were performed without the separation of the water layer.

#### 2.1.2. Purification of Mussel Extracts

Purification of the mussel sample extract is important prior to the analysis. The removal of proteins and DNA lengthens the column lifetime, prevents the blockage of chromatographic system, and decreases the contamination of the mass detector. In the official pre-column oxidation HPLC-FLD method C18 SPE is used for the purification of extracted samples, whereas in the post-column oxidation method easier trichloroacetic acid (TCA) precipitation is used for the removal of proteins. Hydrophilic interaction liquid chromatography (HILIC) based polyhydroxyethyl aspartamide (PHEA) SPE cartridge was applied in the purification of mussel extracts before the PSP analyses in the LC-MS/MS method developed by Dell’Aversano *et al.* [13]. Other type of SPE cartridges have also been utilized in the SPE purification of mussel extracts for LC-MS/MS analysis [10,12,16].

Within this study, SPE purifications of the mussel sample extracts for LC-MS/MS analysis were compared. Acetic acid and hydrochloric acid extracts were purified with the C18 SPE, and the acetonitrile extract obtained without separation of the water layer was purified with two separate HILIC SPE (Table 2, Figure 1). The recovery with hydrochloric acid (26 ng/g) was only about 20% and it was much lower than the recovery with acetic acid extraction (119 ng/g), and this may be due to the suppression effect of chlorine in the MS analyses. Similar effects have also been reported previously by Turrell *et al.* [10]. Despite the solvent exchange from water to LC-MS/MS eluent after the C18 SPE purification, the retention time shift of STX in samples was about 0.2–0.5 min compared to the retention time of STX in reference standard. Thus, these results were not acceptable for the unambiguous identification of STX in the purified mussel extract. Interestingly, when the same C18 purified extract was further diluted 1:10 in the LC-MS/MS eluent and filtered, the retention times were within the acceptable limit and the recovery of STX increased from 119 ng/g to 140 ng/g perhaps due to the decrease of the matrix suppression effect. The retention times of STX in acetonitrile extracts were also within the limits after the HILIC purification and sample dilution into LC-MS/MS eluent. When the SPE purifications had been optimized, the recoveries for STX in acetic acid and acetonitrile extracts were quite similar and close to the value defined for STX obtained by the pre-column oxidation HPLC-FLD method (126–131 ng/g). 

**Table 2:** Preliminary results of the saxitoxin (STX) concentration determination in mussel sample using different extraction solvents and solid phase extraction (SPE) purification procedures. Dilution with liquid chromatography-tandem mass spectrometry (LC-MS/MS) eluent A-B 40:60 (4 mM ammonium formate in H_2_O-ACN, pH 3.5 adjusted with formic acid) and filtration with a syringe filter (PTFE, Millex LCR, 0.45 µm, Ø 13 mm). The results were quantified with external STX standards.

Measured STX conc. (ng/g mussel)	Extraction method	SPE purification	*rt* criterion [26]
119	1% AcOH	C18 SPE, solvent exchange	∆*rt* > 0.2 min
26	0.1 M HCl	C18 SPE, solvent exchange	∆*rt* > 0.2 min
140	1% AcOH	C18 SPE, diluted 1:10, filtered	OK
156	80% ACN, 0.1% formic acid, no heating	PHEA SPE, diluted 1:10	OK
121	80% ACN, 0.1% formic acid, no heating	Si-1 silica SPE, diluted 1:10	OK

#### 2.1.3. LC-MS/MS analysis of STX in Mussel Extracts

The sample preparation for LC-MS/MS was optimized further for original mussel extracts. The samples extracted in 1% acetic acid were centrifuged and then directly diluted (1:10) with the LC-MS/MS eluent containing 60% acetonitrile. The turbid solution formed in the acetic acid extract was filtered with a syringe filter (PTFE, 0.45 µm) prior to the LC-MS/MS analysis. In these experiments, the solvent of the sample was not exchanged, but the samples were diluted in the LC-MS/MS eluent before the PSP analysis. At first, the sample dilution 1:5 with LC-MS/MS eluent was tested, but the retention time shift was more than 0.2 min. Repeatable results were obtained with 1:10 and 1:20 dilutions in LC eluent, and the retention times for STX met the acceptance criteria (|∆*rt*| ≤ 0.2 min). The qualitative identification of STX was also confirmed by the fragmentation pattern of the protonated molecule ion (MS^2^ of [M + H]^+^ at *m/z* 300) identical to the fragment spectrum of the certified reference standard. Acetonitrile extracts fulfilled also the identification criteria when diluted 1:10 with LC-MS/MS eluent, but acetic acid was preferred because of the similarity to the solvent applied in the official pre-column oxidation HPLC-FLD method. The quantitative results were again calculated using an external STX reference standard (Table 3). A slight suppression effect could be still detected and the results were lower than the assigned value (126 ng/g). 

**Table 3:** LC-MS/MS measurements (*n* = 4–5) of diluted mussel extract without SPE purification. Dilution with LC-MS/MS eluent A–B 40:60 (4 mM ammonium formate in H_2_O-ACN, pH 3.5 adjusted with formic acid). Filtration with a syringe filter (0.45 µm). The results were quantified with external STX standards.

Measured STX conc. (ng/g mussel)	Extraction method	Comments	*rt* criterion [26]
103 ± 13 (*n* = 4)	1% AcOH	dilution 1:10, filtration	OK
96 ± 5 (*n* = 5)	80% ACN, 0.1% formic acid, no heating	dilution 1:10	OK

As a conclusion, the dilution of samples before the LC-MS/MS analysis is a fast solution to decrease ion suppression in the mass detector to recover reproducible identification for STX in mussel samples both from 80% ACN and acetic acid extracts. However, due to the difficult sample matrix, accurate quantifications were performed with the standard added into the toxic sample extract in the method applications presented in the following chapter. Comparison between 1% AcOH and 80% acetonitrile showed slightly better or similar yields for 1% AcOH without SPE purification. Due to the similarity to the official method 1% AcOH was chosen as an extracting solvent.

### 2.2. Method Applications

#### 2.2.1. Quantification of STX in Mussel Samples

The developed LC-MS/MS method was applied for the analysis of homogenized mussel samples before the PT (*n* = 3) and for stability studies during the PT (*n* = 6). Further, one set of shipped PT samples was also tested (*n* = 3). Accurately weighed mussel samples (5.0 g) were extracted in 1% acetic acid (10.0 mL), diluted, filtered and analysed with LC-MS/MS. The quantification was performed by adding a reference standard solution (0–25 ng/mL) into the sample extracts. The homogeneity of the mussel samples had been tested prior to the LC-MS/MS method development, and randomly selected samples (*n* = 10) were analysed for STX, dcSTX, neosaxitoxin (NEO), GTX2, GTX3, GTX1, GTX4, and GTX5 from the series of 160 parallel homogenized mussel samples using a pre-column oxidation HPLC-FLD method [5]. The relative standard deviation (RSD) of the PSP toxins in mussel samples was 4.2%–7%, and for STX, 4.8%.

#### 2.2.2. Stability Studies of Proficiency Test (PT) Samples

**Figure 2:**
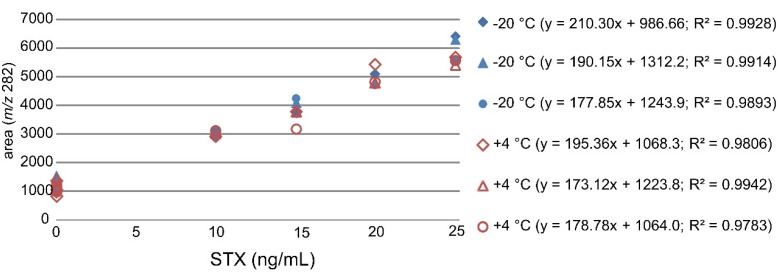
Linear curves of the standard addition method used for quantification of STX in mussel samples stored at −20 °C and +4 °C with three zero points and four STX addition points (10–25 ng/mL).

The stability of STX in mussel samples was studied during the PT. Three mussel samples were stored at +4 °C and three reference samples from the same set of the homogenized mussel samples were stored at −20 °C for four weeks. The samples were extracted and analysed in the same sequence after the four weeks stability study with STX standard addition of 0–25 ng/mL. The standard addition curves displayed good linearities with *R*^2^ values between 0.98 and 0.99 (Figure 2).

The stability test results obtained with the standard addition method were compared with the mussel sample PT results and mussel sample results measured before the PT with the developed LC-MS/MS method (Table 4). The results were all similar and very close to the assigned value measured with the HPLC-FLD method (126 ng/g mussel). The retention times were repeatable and within the limits (|∆*rt*| ≤ 0.2 min).

**Table 4:** The recoveries of STX with the standard addition method from diluted and filtered AcOH extracts analysed with LC-MS/MS (*n* = 3). The errors were estimated from the variation of x-intercept.

Samples	Measured STX conc. ng/mL	Calculated STX, ng/g mussel	*rt* criterion [26]
Mussel sample analyzed before PT	6.0 ± 1.5	120	OK
Mussel sample stability at −20 °C	6.2 ± 1.3	124	OK
Mussel sample stability at +4 °C	6.2 ± 0.8	123	OK
PT mussel samples	6.0 ± 0.7	120	OK

#### 2.2.3. Identification Criteria for STX in Mussel Samples 

**Figure 3:**
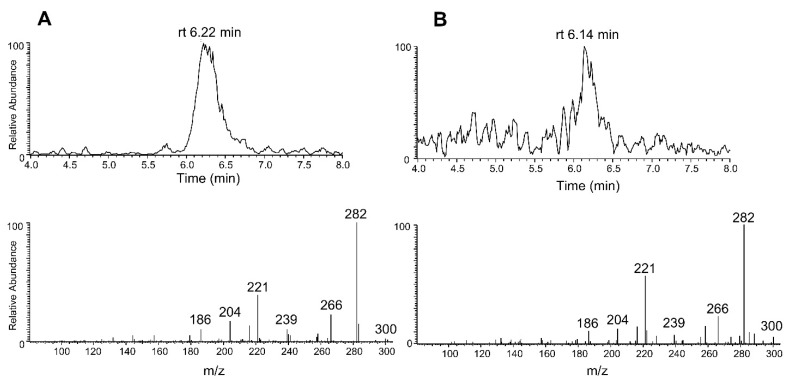
(**A**) Ion chromatogram and LC-MS/MS spectrum of STX standard (10 ng/mL, *rt* = 6.22 min). Signal to noise 70. (**B**) Ion chromatogram and LC-MS/MS spectrum of identified STX in mussel sample (*rt* = 6.14 min). ([M + H]^+^ at *m/z* 300, typical product ions at *m/z* 282, 266, 239, 221, 204, 186 for STX). Signal to noise 13.

The qualitative identification of STX in mussel samples was based on the retention times and relative ion intensities in the mass spectra. LC-MS/MS spectra of STX standard solution (10 ng/mL) and mussel sample extract are presented in Figure 3A and Figure 3B, respectively. The retention time differences were within the required criterion |Δ*rt*| ≤ 0.2 min [26].

The mass fragmentation results were compared to EU criteria [27], which require that the allowed relative mass fragmentation tolerances have to be between 20% and 50% depending on the relative ion intensities. The smaller the relative signal intensity, the larger is the allowed tolerance. Typical fragment ions at *m/z* 282, 266, 239, 221, 204, 186 for STX (MS^2^ [M + H]^+^ at *m/z* 300) were of same relative intensity in the STX standard solution and in the mussel extract, though the smallest fragment ions at *m/z* 239 and 186 were not detectable in all measurements of diluted sample extracts (Table 5). When the optimized LC-MS/MS method was used, the measured results were within the required limits for the reliable identification of STX in mussel sample.

**Table 5:** Identification of STX in mussel samples, retention times (*rt*) and relative ion intensities (ratio of qualifier/Quantifier ions; q/Q) compared to the reference standard, relative ion intensity (maximum allowed relative tolerance): >50% (±20%); >20% to 50% (±25%); >10% to 20% (±30%); ≤10% (±50%) [27].

STX, MS^2^ at *m/z* 300	*rt* (min)	q/Q (%)				
		266/282	239/282	221/282	204/282	186/282
STX reference standard (10 ng/mL, *n* = 5)	6.27 ± 0.03	20 ± 1	9 ± 2	35 ± 3	20 ± 3	9 ± 2
Mussel samples (*n* = 3)	6.16 ± 0.06	20 ± 5	8 ± 1	36 ± 6	19 ± 4	9 ± 2

#### 2.2.4. Identification Criteria for Other PSP Toxins in Mussel Samples 

On the basis of initial HPLC-FLD measurements GTX1&GTX4, GTX2&GTX3, GTX5, dcSTX, and NEO were found to be present in the mussel sample. All these compounds, except GTX4 and GTX5, could be detected in the SPE purified samples during method development. However, due to the higher detection limits for gonyautoxins, GTX1 and GTX3 were not detected in the diluted samples with the above described LC-MS/MS method. Further, GTX5 was not analyzed due to the occasional unavailability of the reference standard. The summary of the identified STX analogues in PT samples is presented in Table 6.

**Table 6:** Identification of other PSP analogues in 1:10 diluted mussel sample extract (number of LC-MS/MS measurements = 3–6).

PSP analogue	[M + H]^+^	Precursor	Standard *rt* (min)	Sample *rt* (min)
GTX1	412	332	4.52 ± 0.01	n.d
GTX2	396	316	4.48 ± 0.02	4.44 ± 0.02
GTX3	396	396	4.77 ± 0.00	n.d.
GTX4	412	412	n.d.	n.d.
GTX5	380	380	n.a.	-
dcSTX	257	257	6.50 ± 0.02	6.41 ± 0.03
NEO	316	316	6.52 ± 0.02	6.44 ± 0.02

#### 2.2.5. Quantitative PT Results of STX, dcSTX and NEO in Mussel Samples

Ten laboratories, of which six laboratories from European Union countries, participated in the STX PT. Six laboratories provided STX results on mussel samples and five laboratories provided dcSTX and NEO results for three parallel mussel samples and triplicate measurements analyzed with pre-column oxidation HPLC-FLD, post-column oxidation HPLD-FLD, or LC-MS/MS methods. The assigned values were measured before the PT with pre-column oxidation HPLC-FLD method and the z-scores were derived from the results provided by the participating laboratories. The optimized LC-MS/MS method was used in the PT for mussel samples and STX, dcSTX, and NEO were quantified in PT samples (*n* = 3) using the standard addition method (0–25 ng/mL). The detection limits of dcSTX and NEO were at about the same level as the detection limit for STX (~1 ng/mL, ~20 ng/g mussel), and these analogues could be quantified in the mussel samples with the quantitation limit of approximately 3 ng/mL and 60 ng/g mussel. The results for STX, dcSTX, and NEO in the PT were acceptable and in good accordance with the assigned values obtained with pre-column oxidation HPLC-FLD (Table 7) [28].

**Table 7:** PT results and z-scores for STX, decarbamoyl saxitoxin (dcSTX), and neosaxitoxin (NEO) in three parallel mussel samples. The results were measured with standard addition into the diluted mussel extracts (0–25 ng/mL, five-point curves). The assigned values were measured with pre-column oxidation HPLC-FLD.

Sample	STX	mussel ng/g	z-score	dcSTX	mussel ng/g	z-score	NEO	mussel ng/g	z-score
	measured	assigned		measured	assigned		measured	assigned	
1	78	126	−1.48	204	194	+0.20	52	55	−0.19
2	134	126	+0.26	195	194	+0.02	72	55	+1.22
3	110	126	−0.50	215	194	+0.42	68	55	+0.98

## 3. Experimental Section 

### 3.1. Materials

Certified reference standard solutions of PSP toxins were purchased from the National Research Council (NRC, Halifax, NS, Canada). The external standards were prepared by diluting the original standard solutions with LC-MS/MS eluent (4 mM ammonium formate-ACN 40:60, pH 3.5 adjusted with formic acid). All solvents used for chromatography were of HPLC grade.

Homogenized mussel samples (*n* = 100) were obtained from Marine Institute (Galway, Ireland) in October 2012 [29]. Toxic Spanish *Mytilus galloprovincialis* whole flesh tissue (500 g), Toxic Canadian *Mytilus edulis* whole flesh tissue (7 g), and blank Irish *Mytilus edulis* mussel whole flesh tissue (893 g) were blended with stabilizers and antibiotics (0.02% of each ethoxyquin, ampicillin, erythromycin, and oxytetracycline) and the moisture content was adjusted to approximately 83.5% with water. The mussel tissues were homogenized with Polytron^®^ 6100 (Kinematica™, Luzern, Switzerland) and dispensed into 5 mL polypropylene tubes (Teklab Ltd., Durham, UK) with a peristaltic pump (Manostat, Barrington, IL, USA) adjusted to dispense approximately 5.3 g homogenized mussel sample. The tubes were purged with nitrogen and hermetically sealed with aluminium seal closures (Seal-it-systems Ltd., Accrington, UK). Wadded screw caps were placed on the tubes. The frozen mussel samples were shipped to VERIFIN (University of Helsinki, Helsinki, Finland), and the samples were stored in a freezer at −20 °C before their use for the stability and proficiency tests.

### 3.2. Sample Preparation 

#### 3.2.1. Extraction with 1% AcOH or 0.1 M HCl 

The mussel samples were extracted with slightly modified AOAC methods [5]. The mussel sample (5.0 g) was extracted with 4 mL of 1% acetic acid or 0.1 M HCl in MilliQ water by shaking the sample with Heidolph Multi Reax mixer (Schwabach, Germany) at room temperature for 30 min. The sample was heated in boiling water at 95–100 °C for 5 min and mixed with Multi Reax mixer for 5 min at room temperature. The sample was cooled in an ice bath for 5 min and centrifuged with 5000 rpm (relative centrifugal force, RCF, 2400× *g*) for 10 min at 4 °C. The supernatant was decanted and the extraction was repeated with another 4 mL of 1% acetic acid or 0.1 M HCl in MilliQ water. Both extraction solvents were combined and the volume was adjusted to 10.0 mL with 1% acetic acid or 0.1 M HCl. The sample extracts were transferred into a centrifuge filter (PVDF Ultrafree MC, 0.45 µm, 0.5 mL, Millipore, Carrigtwohill, Ireland), and centrifuged with 14000 rpm (RCF 21500× *g*) for 5 min at 4 °C. 

#### 3.2.2. C18 SPE Purification of the Mussel Extracts

A total of 1% AcOH or 0.1 M HCl extracted mussel sample was further purified with C18 SPE cartridge (Varian Bond elute C18, 500 mg, Lake Forest, CA, USA). The cartridge was conditioned with 6 mL methanol and 6 mL water. A 1.0 mL aliquot of the crude extract was loaded, and the PSP toxins were eluted with water (2 × 2 mL), and the volume was adjusted to 5.0 mL. The solvent was evaporated under nitrogen flow and exchanged to LC-MS/MS eluent (4 mM ammonium formate-ACN 40:60, pH 3.5 adjusted with formic acid) prior to LC-MS/MS analysis. 

#### 3.2.3. Acetonitrile Precipitation of Acetic Acid Extract

For the LC-MS/MS analyses, crude 1% acetic acid extracted samples were diluted with 1:10 with LC-MS/MS eluent (4 mM ammonium formate-ACN 40:60, pH 3.5 adjusted with formic acid). The precipitate was filtered through a syringe filter (PTFE, 0.45 µm, Ø13 mm, Millex) prior to the LC-MS/MS analysis.

#### 3.2.4. Extraction with 80% Acetonitrile-Water in 0.1% Formic Acid

The mussel samples were extracted with a slightly modified method by Dell’Aversano *et al.* [13]. The mussel sample (5.0 g) was extracted with 4 mL of acetonitrile/water (80:20, *v/v*) containing 0.1% of formic acid. The sample was shaken with Multi Reax mixer (Schwabach, Germany) at room temperature for 30 min and centrifuged with at 5000 rpm (RCF 2400× *g*) for 10 minutes at 4 °C. The supernatant was decanted and the extraction was repeated with another 4 mL of acetonitrile/water (80:20, *v/v*) containing with 0.1% of formic acid. The extraction solvents were combined and the volume was adjusted to 10.0 mL with acetonitrile/water (80:20, *v/v*) containing 0.1% of formic acid. The sample was transferred into a centrifuge filter (PVDF Ultrafree MC, 0.45 µm, 0.5 mL, Millipore, Carrigtwohill, Ireland), and centrifuged with 14000 rpm (RCF 21500× *g*) for 5 min at 4 °C. For the LC-MS/MS analyses, the samples were diluted with 1:10 with LC-MS/MS eluent (4 mM ammonium formate-ACN 40:60, pH 3.5 adjusted with formic acid).

#### 3.2.5. HILIC SPE Purification of the Mussel Samples Extracted in Acetonitrile/Water 80:20 (*v/v*) with 0.1% Formic Acid [13]

The ACN extracted sample was further purified with a PolyHydroxyEthyl Aspartamide^®^ (PHEA) SPE cartridge (SPE HY 2001 series #102810-1-1, PolyLC Inc., Columbia, MD, USA) or Strata-Si-1 Silica cartridge (55 µm, 70A, 500 mg, 6 mL, Phenomenex, Torrance, CA, USA). The cartridge was conditioned with 5 mL of acetonitrile/water (10:90, *v/v*) containing 0.1% formic acid and 5 mL of acetonitrile/water (90:10, *v/v*) containing 0.1% formic acid. A 1.0 mL aliquot of the crude extract was loaded, followed by a washing step with 0.5 mL acetonitrile/water 80:20 (*v/v*) containing 0.1% formic acid. The PSP toxins were eluted into a final volume of 3.0 mL in volumetric tube with acetonitrile/water 10:90 (*v/v*). An additional 2.0 mL of the eluent was collected separately. The wash effluent and the additional 2 mL eluent fraction did not contain STX, and they were discarded.

### 3.3. Instrumentation

#### 3.3.1. LC-MS/MS Method

LC-MS/MS was performed with a Finnigan LXQ liner ion trap mass spectrometer with positive mode electrospray ionization (ESI) source interfaced to a Finnigan Surveyor Autosampler Plus Liquid Chromatograph (ThermoFinnigan, Hemel Hempstead, UK) with a slightly modified method described by Halme *et al.* [25]. The chromatographic separation was carried out on a TOSOH Bioscience 3 µm HILIC TSK-gel Amide-80^®^ column (150 mm × 4.6 mm, Stuttgart, Germany). A mobile phase was 4 mM ammonium formate in H_2_O-ACN 40:60 (*v/v*), and the pH of the eluents was adjusted to 3.5 with formic acid. The flow rate was 1 mL/min with an accurate post-column splitter (1:20) between LC and MS. Spray voltage of 5 kV was applied and nitrogen was used as sheath gas. Capillary temperature was set to 350 °C and the relative collision energy was 29%. STX, dcSTX, and NEO were measured with [M + H]^+^ precursor ions MS^2^ at *m/z* 300, *m/z* 257 and *m/z* 316 for STX, dcSTX and NEO, respectively. The quantification was based on the signal area of the quantifier (Q) ions at *m/z* 282, *m/z* 239, and *m/z* 298 for STX, dcSTX and NEO, respectively.

#### 3.3.2. HPLC-FLD Method 

All measurements were based on the procedures given in the AOAC Official Method 2005.06. [5] An HPLC system from Thermo Fisher Scientific (Reinach, Switzerland) was used with the following components: UltiMate LPG-3400RS quaternary analytical pump, UltiMate 3000 analytical split-loop autosampler, UltiMate TCC-3000RS column thermostat and a fluorescence detector RF-2000 with excitation wavelength at 340 nm and emission wavelength at 400 nm. The chromatographic conditions were chosen according to AOAC [5]. A Supelcosil C18 column (150 × 4.6 mm, 5 µm particle size) from Sigma-Aldrich Chemie GmbH (Buchs, Switzerland) was used. The eluent consisted of 2 phases: A: 0.1 M ammonium formate (5% acetonitrile), B: 0.1 M ammonium formate. Flow rate was 2 mL/min. The gradient conditions were: initially 0% A, hold for 1 minute, increase to 5% in 4 min, increase 5%–70% in 5 min, hold for 2 min and return to 0% A and *re*-equilibrate for 5 min. The column temperature was kept at 25 °C.

## 4. Conclusions 

As a summary, various sample preparation methods were studied for the analysis of STX in mussel samples with LC-MS/MS. It was found that several factors influence the identification and quantification of STX in mussel matrix using LC-MS/MS.

The sample preparation methods were based on the official methods developed for the analysis of PSP toxins in mussel samples. The best recovery for STX was obtained with 1% acetic acid extraction of mussel material. Though hydrochloric acid is commonly accepted for several techniques, it was found to be an unsuitable extraction solvent for mussel samples analysed by LC-MS/MS, mainly because of the strong matrix suppression effect. Various purification methods for mussel extracts were tested and finally a sample dilution with an acetonitrile precipitation step turned out to be optimal for the purification of the acetic acid extracts for LC-MS/MS analysis. 

The best quantification results were obtained with the standard addition method where a standard solution of STX (0–25 ng/mL) was added into the mussel extracts before filtration and analysis by LC-MS/MS. The identification criteria defined for STX met the applied criteria of retention time shift and mass fragmentation tolerances. 

The developed method was applied in the STX PT for the identification and quantification of STX, dcSTX, and NEO, and the results were in good agreement with the assigned values measured with the pre-column oxidation HPLC-FLD method. 

Compared to other PSP toxin identification methods, the LC-MS/MS method is superior with respect to the unambiguous identification of the analyte due to the combination of chromatographic data and mass spectral information. The LC-MS/MS method should thus be thoroughly validated to become an additional official method for the analysis of PSP toxins in mussel samples.
